# Comparative Transcriptome Profiling Reveals Compatible and Incompatible Patterns of Potato Toward *Phytophthora infestans*

**DOI:** 10.1534/g3.119.400818

**Published:** 2019-12-09

**Authors:** Yanfeng Duan, Shaoguang Duan, Miles R. Armstrong, Jianfei Xu, Jiayi Zheng, Jun Hu, Xinwei Chen, Ingo Hein, Guangcun Li, Liping Jin

**Affiliations:** * Institute of Vegetables and Flowers, Chinese Academy of Agricultural Sciences; Key Laboratory of Biology and Genetic Improvement of Tuber and Root Crop, Ministry of Agriculture and Rural Affairs, Beijing 100081, China,; † The University of Dundee, Division of Plant Sciences at the James Hutton Institute, Dundee, DD2 5DA, UK, and; ‡ Cell and Molecular Sciences, The James Hutton Institute, Dundee, DD2 5DA, UK

**Keywords:** Potato, late blight, *Phytophthora infestans*, RNAseq, dRenSeq

## Abstract

Late blight, caused by *Phytophthora infestans* (*P. infestans*), is a devastating disease in potato worldwide. Our previous study revealed that the *Solanum andigena* genotype 03112-233 is resistant to *P. infestans* isolate 90128, but susceptible to the super race isolate, CN152. In this study, we confirmed by diagnostic resistance gene enrichment sequencing (dRenSeq) that the resistance of 03112-233 toward 90128 is most likely based on a distinct new *R* gene(s). To gain an insight into the mechanism that governs resistance or susceptibility in 03112-223, comparative transcriptomic profiling analysis based on RNAseq was initiated. Changes in transcription at two time points (24 h and 72 h) after inoculation with isolates 90128 or CN152 were analyzed. A total of 8,881 and 7,209 genes were differentially expressed in response to 90128 and CN152, respectively, and 1,083 differentially expressed genes (DEGs) were common to both time points and isolates. A substantial number of genes were differentially expressed in an isolate-specific manner with 3,837 genes showing induction or suppression following infection with 90128 and 2,165 genes induced or suppressed after colonization by CN152. Hierarchical clustering analysis suggested that isolates with different virulence profiles can induce different defense responses at different time points. Further analysis revealed that the compatible interaction caused higher induction of susceptibility genes such as *SWEET* compared with the incompatible interaction. The salicylic acid, jasmonic acid, and abscisic acid mediated signaling pathways were involved in the response against both isolates, while ethylene and brassinosteroids mediated defense pathways were suppressed. Our results provide a valuable resource for understanding the interactions between *P. infestans* and potato.

Potato (*Solanum tuberosum* L.) is the world’s most important non-cereal food crop and ranks third in terms of food production (Slater *et al.* 2014; Lenman *et al.* 2016). Late blight disease is caused by the oomycete pathogen *Phytophthora infestans*, which was responsible for the Irish famine in the 1840s. More than 170 years later, late blight remains the most significant threat to potato production worldwide (Gao *et al.* 2013), resulting in 16% yield losses globally (Sanju *et al.* 2015). The pathogen infects the entire plant, including stems, leaves and tubers (Fry 2008), and can completely destroy infected potato fields in a matter of days (Lenman *et al.* 2016). Current disease management practices rely on frequent fungicide applications (Orbegozo *et al.* 2016), which are of environmental concern (Haas *et al.* 2009). In addition, effective chemicals are not always readily available and/or are too costly for farmers in developing countries, exacerbating the threat of late blight to income and food security. Globally, costs associated with chemical control and crop losses amount to at least €5.6 billion per year (Ali *et al.* 2014).

Deployment of genetic resistance to combat pests and diseases is considered to be the most cost-effective and environment-benign strategy for crop protection (Michelmore *et al.* 2013). To date, over 20 resistance (*R*-) genes conferring resistance against potato late blight have been cloned, and all belong to the nucleotide-binding, leucine-rich-repeat (NLR) class (Jo *et al.* 2015). Many of these *R*-genes have been successfully transferred into cultivated potato (Frades *et al.* 2015). However, *R*-gene-mediated resistance (known as qualitative or major resistance) has been rapidly overcome by fast-evolving *P. infestans* isolates (Khavkin 2015). Quantitative resistance, usually controlled by multiple genes with minor effects, is believed to be more durable against *P. infestans* (Colton *et al.* 2006). Nevertheless, quantitative resistance is often influenced by environmental conditions (Jo *et al.* 2015), and its multigenic nature makes it difficult to introgress into varieties through conventional breeding (Draffehn *et al.* 2013). Furthermore, a recent study has shown that the major dominant potato *R*-gene *R8* can also exhibit a variable resistance phenotype that is typically associated with quantitative resistance (Jiang *et al.* 2018). In conclusion, although there has been some limited success in controlling late blight by introducing qualitative or quantitative resistances into potato cultivars through breeding (van der Lee *et al.* 2001), knowledge of the mechanisms of resistance is lacking.

Understanding plant resistance mechanisms is important for developing complimentary strategies for disease control. Plants have a complex immune system that provides two lines of defense with different molecular mechanisms of pathogen recognition (Jones and Dangl 2006; Wang *et al.* 2018). The first line of defense is referred to as pathogen-associated molecular pattern (PAMP)-triggered immunity (PTI), in which PAMPs are recognized by pattern recognition receptors (PPRs) that often have a kinase domain (Tariq *et al.* 2018). However, successful pathogens have evolved effectors that interfere with PTI responses, enabling successful infection, and this is known as effector-triggered susceptibility (ETS) (Jones and Dangl 2006; Burra *et al.* 2018). The second line of defense, termed effector-triggered immunity (ETI), is the detection of effector proteins in the host cytoplasm by resistance proteins, which elicits further immunity (Jones and Dangl 2006; Wang *et al.* 2018). Typically, PTI is a response to conserved pathogen molecules, while ETI is highly specific and often leads to programmed cell death (PCD) manifested by the hypersensitive reaction (Khavkin 2015). As complicated as the mechanisms of disease resistance in plants can be, genome-wide expression profiling during pathogen infection can help to identify key components of resistance pathways (Feys and Parker 2000). Although transcriptome dynamics during the interaction between potato and *P. infestans* have been reported (Gao *et al.* 2013; Ali *et al.* 2014; Frades *et al.* 2015; Yang *et al.* 2018), none of these studies investigated the responses of a single host to different isolates with contrasting infection capabilities. In our previous study, we identified two *P. infestans* isolates, 90128 and CN152, that resulted in an incompatible and compatible interaction, respectively, on the *S. andigena* genotype 03112-233 (2n = 4x = 48), which was obtained from the National Research Support Project-6 (NRSP-6) in the United States. The isolate 90128 is virulent on potato differentials carrying the *R*-genes 1, 3, 4, 6, 7, 8, 10, and 11 from *S. demissum* and was isolated in the Netherlands (Vleeshouwers *et al.* 1999). CN152 is virulent on plants carrying the *R*-genes 1, 3b, 4, 5, 6, 7, 8, 9, 10, and 11 and was isolated in Sichuan province in China. The isolate CN152 can overcome many of the known late blight resistance genes including the broad-spectrum resistance gene *RB*, which is also known as *Rpi-blb1*, and is thus considered a ‘super race’ isolate (Yang *et al.* 2018; Li *et al.* 2017). In this study, RNA profiling was performed at two different time points on 03112-233 potato leaves infected by the two contrasting isolates. The objective was to characterize defense responses of a single host against incompatible and compatible late blight isolates and to elucidate the defense pathways involved. The generated transcriptome data can provide valuable insight into compatible and incompatible plant-pathogen interactions.

## Materials and Methods

### Plant materials, P. infestans isolates and treatments

The primitive cultivated potato (*S*. *andigena*) genotype 03112-233 maintained at the Institute of Vegetables and Flowers, Chinese Academy of Agricultural Sciences, China, was used in this study. The genotype was grown in a growth chamber set at 20° with a 16 h/8 h (light/dark) cycle and 70% relative humidity. Two different *P. infestans* isolates were used in this study. One isolate, 90128, induces an incompatible reaction (resistance symptoms) on *S. andigena* 03112-233 leaves, while the other isolate, CN152, induces a compatible reaction (susceptible symptoms). Both isolates were maintained on rye medium at 18°. Freshly produced sporangia were collected in sterile water and incubated at 4° for 3–6 h to release zoospores. For each isolate, nine fully expanded leaves detached from the same 6-week-old plant were inoculated with two 10 μL drops containing 15,000 sporangia/mL, and three biological replicates (plants) were performed for each treatment. The inoculated leaves were kept in a climate chamber at 15° with a 16 h light/8 h dark cycle. Three leaves per treatment were sampled at 24 and 72 hpi, respectively and the remaining leaves were further incubated for 6–7 d after harvest to monitor the success of the inoculations. Uninoculated leaves were used as the controls for the experiment.

Resistance testing was not only performed on detached leaves but also on *in vitro* plantlets inoculated with 90128 and CN152. Resistance scoring was performed as described by van der Lee *et al.* (2001).

### DNA extraction and dRenSeq analysis

Genomic DNA of 03112-233 was extracted according to the modified CTAB procedure of Doyle and Doyle (1987). NLRs enrichment and dRenSeq analysis were conducted as described by Armstrong *et al.* (2018). Paired-end Illumina MiSeq sequencing was used to sequence the *R*-gene enriched samples.

### RNA extraction and library preparation for Illumina sequencing

RNA was extracted from all three biological replicate samples. Total RNA was extracted from inoculated as well as uninoculated leaves using Trizol Reagent (Invitrogen, Carlsbad, CA, USA) according to the manufacturer’s instructions. Removal of genomic DNA was performed using RNase-free DNase I (TaKaRa, Kyoto, Japan). The total concentration of RNA was determined using a NanoDrop microvolume spectrophotometer (Thermo Scientific NanoDrop Products, Waltham, MA, USA). The Illumina HiSeq 4000 platform was used for RNAseq based on PE 150. Library construction and RNAseq were carried out by Novogene Bioinformatics Technology Co., Ltd., Beijing, China.

### Data analysis

The raw image data files were transformed into the original sequenced reads (raw reads) by CASAVA 2.19 base calling analysis (Hu *et al.* 2018) and processed using in-house Perl scripts. Clean data were obtained by removing adapter sequences, poly-N-containing reads and low-quality reads. The DM1-3 516R44 genome sequence (SolTub 3.0) and annotation files were downloaded from the ENSEMBL plants database (ftp://ftp.ensemblgenomes.org/pub/plants/release-37/fasta/solanum_tuberosum/dna/) (Bolser *et al.* 2017). HISAT 2.0.4 was used to align RNAseq reads against the reference genome (Kim *et al.* 2015). HTSeq v0.6.1 was employed to count the reads mapped to each gene (Anders *et al.* 2015). Differential expression analysis between *P. infestans* treated samples and control was performed using DESeq 1.10.1 (Anders and Huber 2010) based on the negative binomial distribution. The resulting *p* values were adjusted using the Benjamini and Hochberg’s approach. Genes with a fold change > 2 and an adjusted *p*-value < 0.05 were defined as significant DEGs. Hierarchical clustering analysis was performed using Cluster 3.0.

### Gene Ontology and Kyoto Encyclopedia of Genes and Genomes enrichment analysis of DEGs

Gene ontology (GO) enrichment analysis of DEGs was performed using GOseq based on a hypergeometric test (Young *et al.* 2010). KOBAS v2.0 was used to test for statistically significant enrichment of DEGs in Kyoto encyclopedia of genes and genomes (KEGG) pathways (Mao *et al.* 2005). All annotated *S. tuberosum* genes in the SolTub 3.0 assembly in ENSEMBL were used as background for GO and KEGG enrichment analysis. GO and KEGG terms with an adjusted *p*-value < 0.05 were considered significantly enriched in DEGs.

### Quantitative RT-PCR analysis

A set of 10 selected DEGs from the transcriptome analysis were validated by qRT-PCR using the same RNA samples that were used for transcriptome analysis. Primers were designed using Primer 5 software (Table S1), and *EIF-3e* was used as the internal control (Kloosterman *et al.* 2013). A total of 1–2 μg of total RNA was used per 20 μL reverse transcription reaction. PCR was performed in a 10 μL reaction mixture with 5 μL SYBR Premix Ex Taq (Takara, Japan), 0.2 μL of both forward and reverse primers, 3.6 μL of double-distilled H_2_O and 1 μL (40 ng/μL) of the cDNA. qRT-PCR was performed using SYBR Green (Bio-Rad) in a Light Cycler 480 System (Roche). The thermal cycler conditions were 95° for 5 min, followed by 40 cycles of 95° for 10 s, 60° for 20 s, and 72° for 20 s. All qRT-PCR experiments were performed in triplicate using independent samples. Relative gene expression was calculated according to the 2^−△△Ct^ method (Livak and Schmittgen 2001).

### Data availability

The raw sequence data have been deposited in the Genome Sequence Archive (Wang *et al.* 2017) in the BIG Data Center (Xu *et al.* 2018), Beijing Institute of Genomics (BIG), Chinese Academy of Sciences, under accession number CRA001418 and are publicly accessible at http://bigd.big.ac.cn/gsa. The supplemental files contain the following data; Table S1 contains qRT-PCR primers for the validation of RNAseq data. Table S2 contains detailed summary of RNAseq results. Table S3 lists 1083 DEGs during both 90128 and CN152 infection. Table S4 to S11 list all isolate-specific DEGs and their gene expression data. Table S12 to S15 list continuously up- or down-regulated genes in the 90128- or CN152-infected samples. Figure S1 contains qRT-PCR based validation of DEGs in response to 90128 and CN152. Supplemental material available at figshare: https://doi.org/10.25387/g3.10298015.

## Results

### dRenSeq analysis reveals that S. andigena genotype 03112-233 contains no known NLR

**Figure 1:**
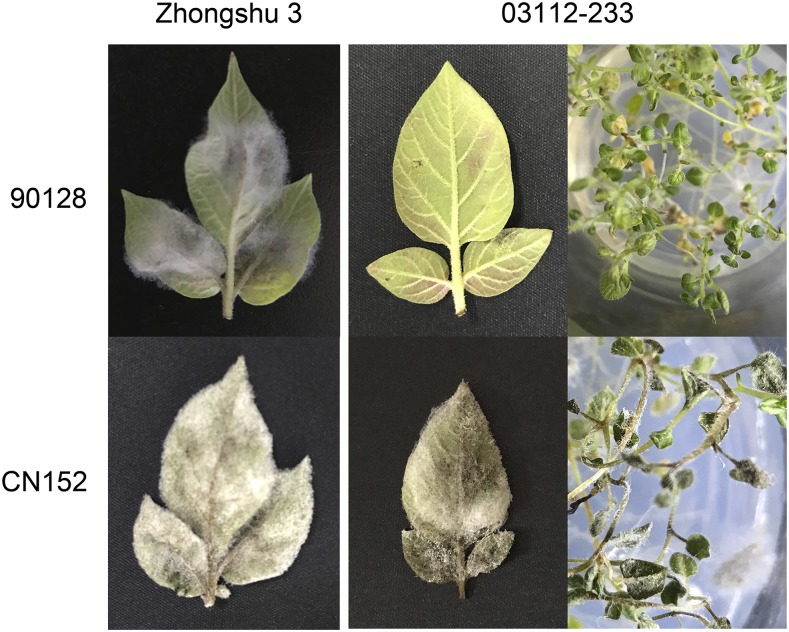
Comparison of detached leaves and *in vitro* plantlets of potato genotype 03112-233 after inoculation with *P. infestans* isolates 90128 and CN152. The control genotype Zhongshu 3 is susceptible to both isolates. Photographs were taken 7 days post inoculation.

The resistance of potato genotype 03112-233 to the *P. infestans* isolates 90128 and CN152 was tested using both detached leaves and *in vitro* plantlets. The results of three independent biological replicates confirmed that 03112-233 is resistant against isolate 90128 and susceptible toward isolate CN152. A control plant, cultivar Zhongshu 3, was susceptible to both isolates, confirming that both isolates were viable (Figure 1).

**Figure 2:**
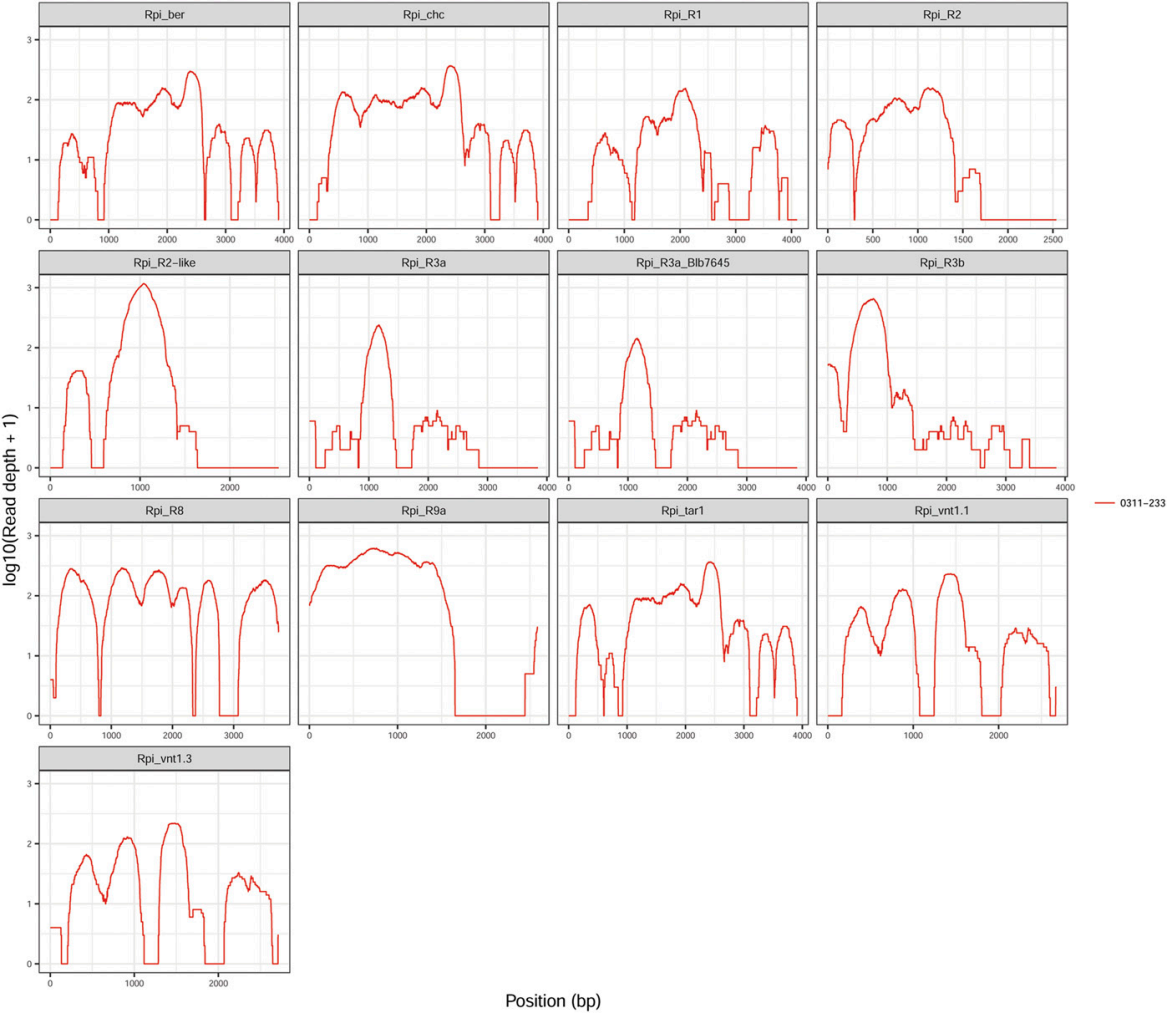
dRenSeq analysis of 03112-233. RenSeq-derived reads were mapped against a reference set of 21 known NLR genes in very-sensitive mode, and the results for 13 NLRs are shown here. Each box represents an entire NLR coding sequence from the start codon to the stop codon (x-axis). The y-axis reveals the coverage of the NLRs on a log scale. Mapping of the reads was carried out at 0% and 2% mismatch rates, and the results for 2% mismatch rate are shown.

To confirm whether the resistance of 03112-233 to *P. infestans* isolate 90128 is caused by known or novel resistance genes, we conducted a dRenSeq analysis (Armstrong *et al.* 2018). RenSeq-enriched paired-end Illumina reads from 03112-233 were mapped at mismatch rates of 0% and 2% against a panel of known functional NLRs: *Rpi_ber*, *Rpi_chc*, *Rpi_R1*, *Rpi_R2*, *Rpi_R2-like*, *Rpi_R3a*, *Rpi_R3b*, *Rpi_R8*, *Rpi_R9a*, *Rpi_tar1*, *Rpi_vnt1.1*, *Rpi_vnt1.3*, *Rpi_Mcq1.1*, *Rpi_Ph-3*, *Rpi_abpt*, *Rpi_amr3*, *Rpi_blb1*, *Rpi_blb2*, *Rpi_blb3*, *Rpi_pta1*, and *Rpi_sto1*. For a gene to be considered ‘present’ within an accession, the sequence had to be identical when compared to the reference gene coding sequence (Strachan *et al.* 2019). Mapping results demonstrated that no known characterized NLR gene was fully represented by the dRenSeq analysis (Table 1, Figure 2), which suggests that the resistance is based on hitherto unknown defense gene(s).

**Table 1:** NLR coverage in 03112-233

Gene name	Percentage of reads mapped to DM target regions at 0% and 2% mismatch rates	
	0%	2%
*Rpi_Mcq1.1*	0.00	58.05
*Rpi_Ph-3*	0.00	5.01
*Rpi_R1*	3.24	76.06
*Rpi_R2*	15.68	66.71
*Rpi_R2-like*	11.64	53.54
*Rpi_R3a*	7.95	62.98
*Rpi_R3b*	12.33	80.97
*Rpi_R8*	28.76	89.78
*Rpi_R9a*	39.74	69.37
*Rpi_abpt*	11.66	56.54
*Rpi_amr3*	0.00	16.07
*Rpi_ber*	15.12	90.20
*Rpi_blb1*	0.00	35.80
*Rpi_blb2*	8.61	78.66
*Rpi_blb3*	0.00	49.10
*Rpi_chc*	17.67	92.48
*Rpi_pta1*	0.00	27.43
*Rpi_sto1*	0.00	14.83
*Rpi_tar1*	10.30	91.85
*Rpi_vnt1.1*	9.38	76.76
*Rpi_vnt1.3*	9.23	80.32

### RNAseq reads aligned well with the potato reference genome sequence

Fifteen sequencing libraries were generated and indexed using total RNA extracted from three independent replicates of (1) healthy leaves, (2) 90218-infected leaves at 24 hpi, (3) 90218-infected leaves at 72 hpi, (4) CN152-infected leaves at 24 hpi and (5) CN152-infected leaves at 72 hpi (Table 2, Table S2). A total of 814,013,764 raw reads were generated using Illumina deep sequencing technology. After the removal of adapter-containing reads, poly-N-containing reads and low-quality reads from the raw data, 801,035,612 high-quality reads (120.16 Gb) were retained. The GC content ranged from 41.25 to 42.58%. To evaluate the reliability of the sequence data, we mapped the clean reads to the doubled monoploid (DM) potato reference genome using HISAT software. The majority of reads, ranging from 78.99 to 86.93%, could be mapped uniquely to the reference genome sequence.

**Table 2:** Summary of Illumina sequencing data

Samples	Raw reads	Clean reads	Clean bases	Total mapped	GC %	Notes
ck_1	63,907,692	62,954,420	9.44 G	56,800,472 (90.22%)	42.58	replicate 1
ck_2	59,612,900	58,987,528	8.85 G	52,618,695 (89.2%)	42.36	replicate 2
ck_3	59,891,922	59,160,746	8.87 G	52,854,685 (89.34%)	42.30	replicate 3
90128_24_1	53,162,668	52,465,884	7.87 G	44,936,121 (85.65%)	41.75	replicate 1
90128_24_2	58,749,064	57,092,568	8.56 G	49,270,027 (86.3%)	41.25	replicate 2
90128_24_3	54,687,216	53,403,434	8.01 G	46,230,121 (86.57%)	41.25	replicate 3
90128_72_1	42,987,040	42,442,160	6.37 G	37,115,818 (87.45%)	42.31	replicate 1
90128_72_2	47,864,298	47,050,222	7.06 G	41,590,631 (88.4%)	42.48	replicate 2
90128_72_3	51,440,666	50,783,440	7.62 G	44,663,116 (87.95%)	42.58	replicate 3
CN152_24_1	51,628,218	50,645,970	7.60 G	44,366,302 (87.6%)	42.02	replicate 1
CN152_24_2	52,733,816	51,609,526	7.74 G	46,095,042 (89.31%)	42.30	replicate 2
CN152_24_3	54,542,236	53,851,118	8.08 G	46,975,999 (87.23%)	42.30	replicate 3
CN152_72_1	50,939,892	50,291,180	7.54 G	44,139,980 (87.77%)	42.39	replicate 1
CN152_72_2	51,514,318	50,912,860	7.64 G	45,624,288 (89.61%)	42.37	replicate 2
CN152_72_3	60,351,818	59,384,556	8.91 G	48,985,802 (82.49%)	42.50	replicate 3
**Total**	**814,013,764**	**801,035,612**	**120.16 G**	**702,267,099 (87.67%)**	**42.18**	**-**

### Patterns of differential gene expression highlight the faster induction of DEGs during an incompatible interaction

**Figure 3:**
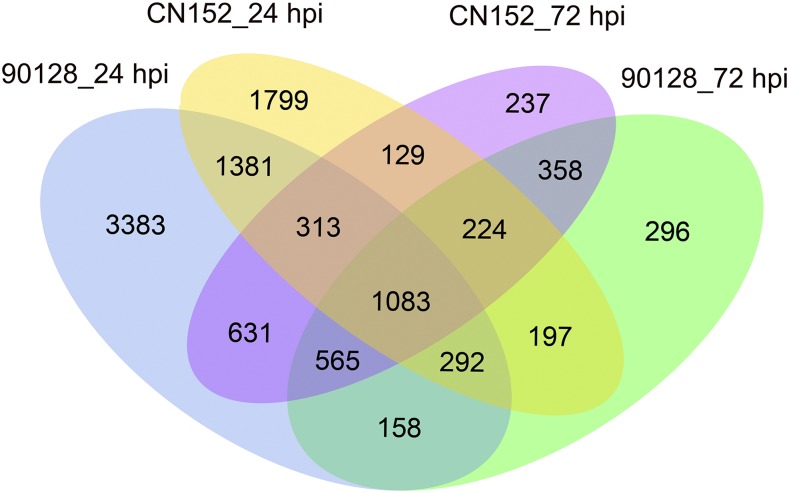
Venn diagram showing the commonalities and differences in the DEGs identified for each *P. infestans* isolate and time point.

To study DEGs in the potato genotype 03112-233 following incompatible and compatible interactions with late blight, gene expression at two time points, 24 hpi and 72 hpi, was assessed and compared with that in uninoculated leaves. An adjusted *p*-value threshold (< 0.05) was set to retrieve the significant DEGs. DEGs were filtered on the basis of a log_2_fold change (log_2_FC) > 1 or < -1. Only the DEGs that met both criteria were retained for further analysis. A total of 11,046 genes were identified as DEGs after 90128 and CN152 infection at 24 hpi and 72 hpi (Figure 3). At 24 hpi, more genes were differentially expressed in response to 90128 (7,806) than CN152 (5,418), while the opposite was observed at 72 hpi (3,173 *vs.* 3,540) (Table 3). Moreover, the number of up-regulated and down-regulated DEGs in response to both 90128 and CN152 decreased at 72 hpi, indicating that the bulk of host DEGs are initiated early (24 hpi) upon pathogen detection and ingress.

**Table 3:** Number of genes differentially regulated in response to 90128 and CN152 in 03112-233

*P. infestans* isolates	24 hpi		72 hpi	
	up	down	up	down
90128	3,888	3,918	1,939	1,234
CN152	3,204	2,214	2,020	1,520

### Hierarchical clustering and KEGG enrichment analysis of DEGs reveal the complexity of host-pathogen interactions

**Figure 4:**
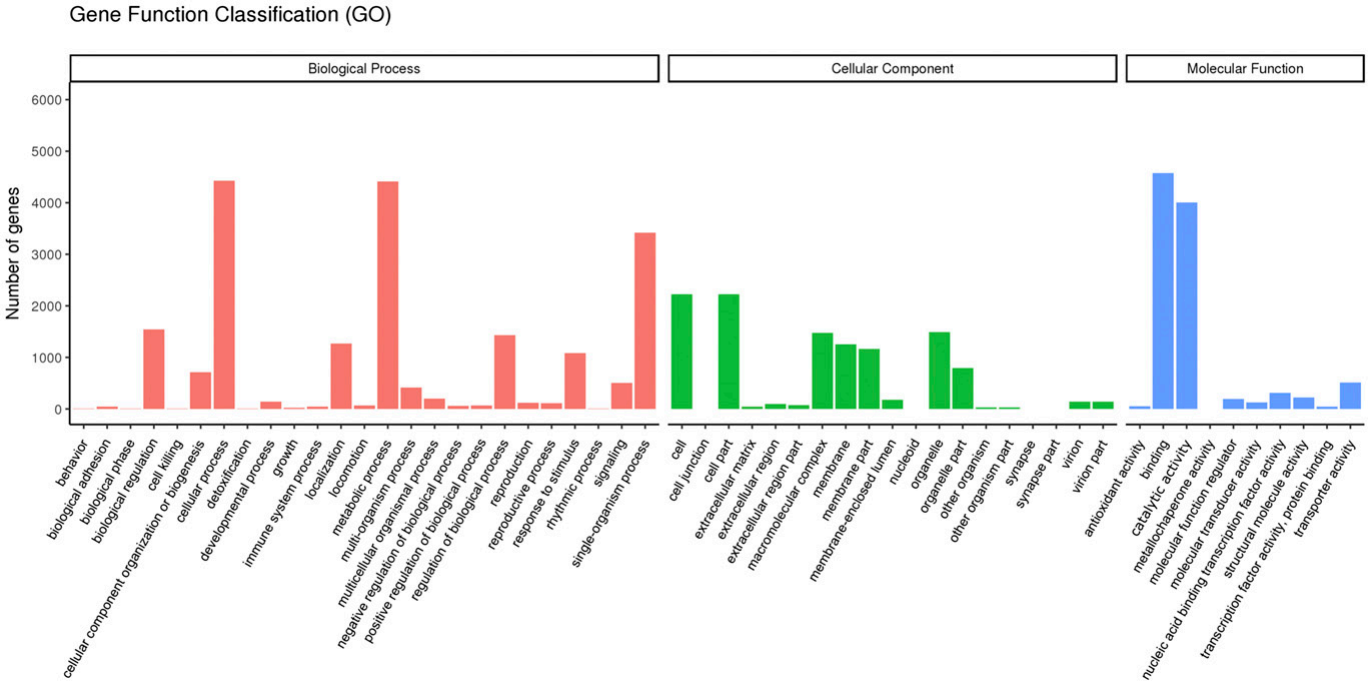
GO classification of annotated DEGs.

Among the 11,046 DEGs, 7,983 (72.27%) genes were annotated in the GO database. To obtain an overview of the putative functions of the genes that participate in the response to *P. infestans* infection, these annotated DEGs were subjected to GO term enrichment analysis. DEGs fell into 54 main groups of enriched GO terms: 25 ‘biological process’, 19 ‘cellular component’, and 10 ‘molecular function’ (Figure 4). For the biological process category, ‘cellular process’ had the most DEGs (4,426), followed by ‘metabolic process’ (4,409 DEGs) and ‘single-organism process’ (3,417 DEGs). For the ‘cellular component category’, most DEGs were annotated to ‘cell’ (2,226 DEGs), ‘cell part’ (2,226 DEGs) and ‘organelle’ (1,488 DEGs). For the ‘molecular function’ category, ‘binding’ had the most DEGs (4,573 DEGs), followed by ‘catalytic activity’ (4,008 DEGs).

**Figure 5:**
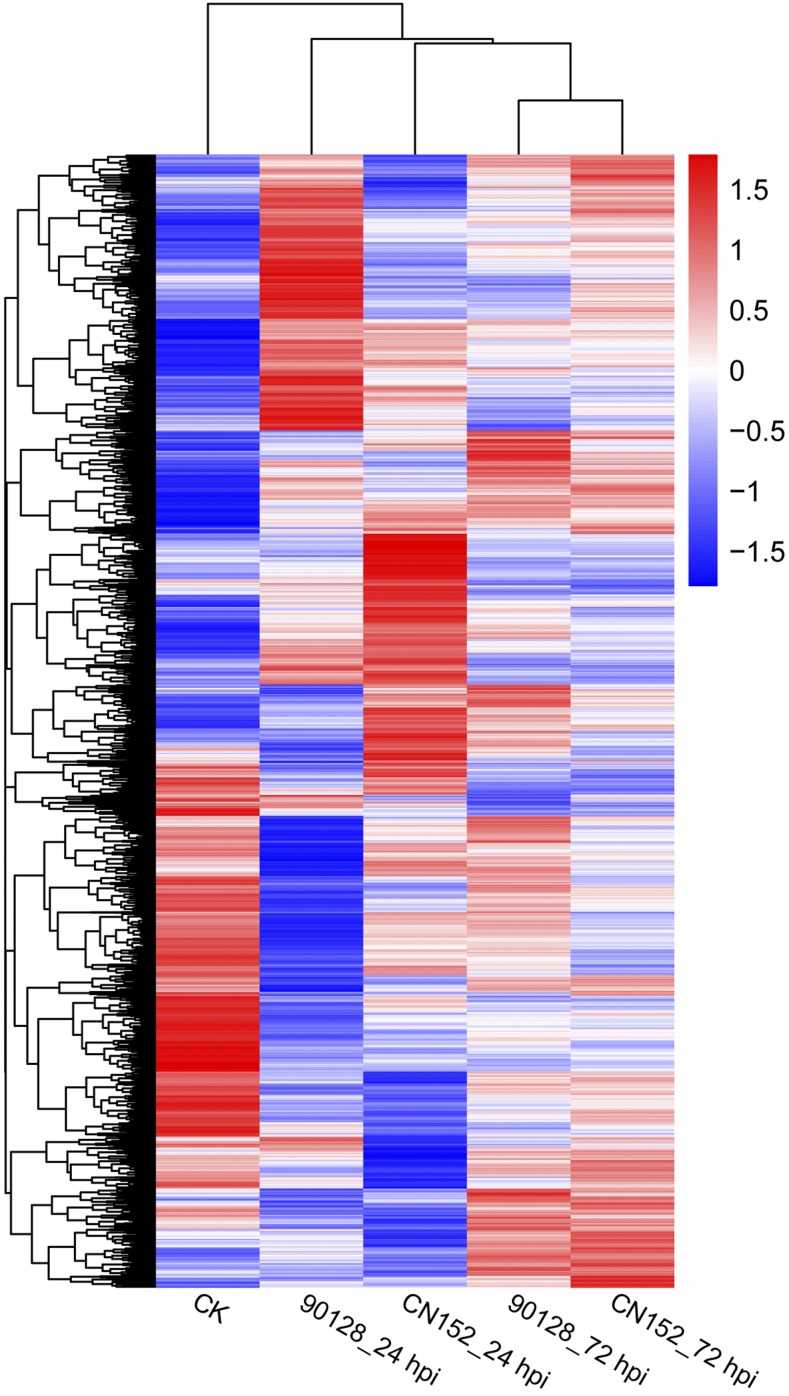
Hierarchical clustering of DEGs identified in the five experimental treatments. Log_10_(FPKM+1) values were used to cluster 11,046 DEGs in Cluster 3.0, and the average values of FPKM from the three biological replicates were used for log_10_(FPKM+1) calculation. Red indicates high expression, and blue indicates low expression.

Hierarchical clustering analysis was employed to investigate the correlation between DEGs in incompatible and compatible interactions at the two time points (24 and 72 hpi). The results revealed how the DEG expression profiles diverge over time and are dependent on the outcome of the host/pathogen interaction (Figure 5). For both the incompatible and compatible interactions, the DEG expression profiles at 24 and 72 hpi were quite different from each other. At 24 hpi, the DEG expression profiles were noticeably different between incompatible and compatible interactions, whereas at 72 hpi they were more similar. This finding indicates that most gene expression changes in the host in response to incompatible or compatible isolates occur at the early stage of infection.

**Figure 6:**
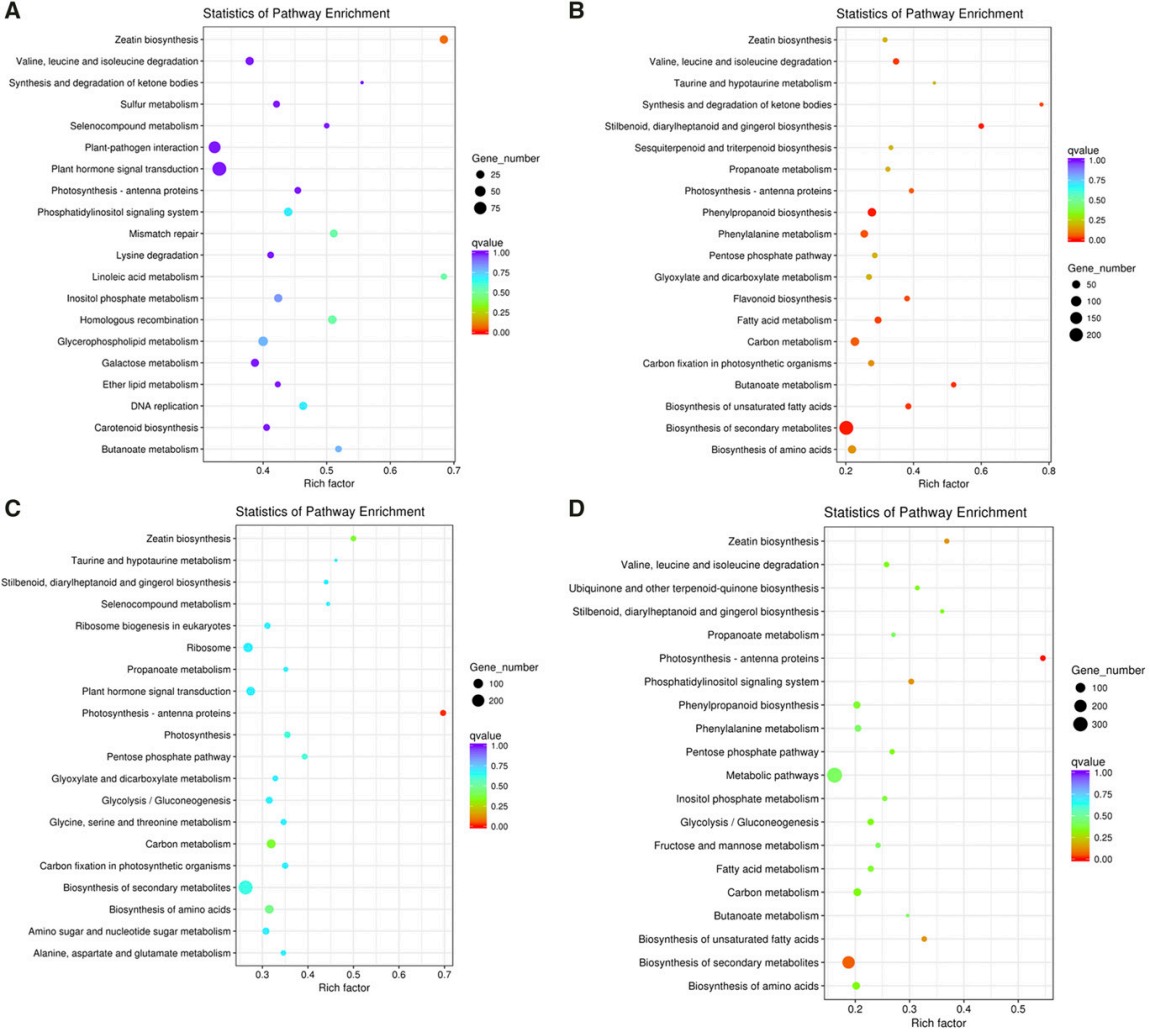
The top 20 KEGG pathways enrichment of DEGs for the 90128-infected samples at 24 hpi (A) and 72 hpi (B) and for the CN152-infected samples at 24 hpi (C) and at 72 hpi (D). The x-axis indicates the rich factor and the y-axis indicates the pathway names. Rich factor refers to the ratio of the number of DEGs located in the KEGG pathway and the total number of genes in the KEGG pathway. The larger the rich factor the greater the degree of enrichment. Significant differences are considered at Benjamini–Hochberg adjusted *p*-value < 0.05.

To identify the pathways activated in response to 90128 and CN152, KEGG pathway enrichment analysis was performed. The top 20 enriched pathways according to adjusted *p*-values are shown in Figure 6. At 24 hpi, the most significantly enriched pathway for DEGs from the incompatible interaction was zeatin biosynthesis, while for the compatible interaction, the pathway photosynthesis - antenna proteins was most significantly enriched. At 72 hpi, the most significantly enriched pathway for both the incompatible and compatible interactions was biosynthesis of secondary metabolites.

### Analysis of shared DEGs reveals common sets of genes involved in the general defense against P. infestans

Genes differentially expressed in response to 90128 and CN152 at 24 and 72 hpi were further analyzed to identify commonalities and differences. As shown in the Venn diagram in Figure 3, 1,083 DEGs were identified in all treatments and time points (Table S3). Of these 1083 shared DEGs, 809 were annotated to 46 enriched GO terms, including ‘response to stimulus’, ‘signaling’, and ‘nucleic acid binding transcription factor activity’, and the top 20 terms are summarized in Table 4. Further analysis showed that 549 shared genes were up-regulated in response to all treatments and at all time points, and 527 genes were down-regulated. One gene encoding a plastid lipid-associated protein was down-regulated at 24 hpi and up-regulated at 72 hpi in both the 90128- and CN152-infected samples. A ribulose bisphosphate carboxylase gene, an uncharacterized LOC102582031 gene and a glycoside hydrolase gene were down-regulated in the CN152-infected samples at 24 hpi and up-regulated at 72 hpi, but all three genes were up-regulated in the 90128-infected samples at 24 and 72 hpi. A raffinose synthase gene, a Myb transcription factor gene and a fatty acyl-CoA reductase gene were up-regulated in the CN152-infected samples at 24 hpi and down-regulated at 72 hpi, but all three genes were down-regulated in the 90128-infected samples.

**Table 4:** The top 20 GO biological process terms for common DEGs in response to 90128 and CN152

Code	GO ID (Lev2)	GO Term (Lev2)	GO Term (Lev1)	Gene Number
1	GO:0008152	metabolic process	Biological Process	476
2	GO:0005488	binding	Molecular Function	467
3	GO:0009987	cellular process	Biological Process	452
4	GO:0003824	catalytic activity	Molecular Function	414
5	GO:0044699	single-organism process	Biological Process	387
6	GO:0005623	cell	Cellular Component	222
7	GO:0044464	cell part	Cellular Component	222
8	GO:0065007	biological regulation	Biological Process	169
9	GO:0032991	macromolecular complex	Cellular Component	152
10	GO:0050789	regulation of biological process	Biological Process	152
11	GO:0043226	organelle	Cellular Component	137
12	GO:0016020	membrane	Cellular Component	131
13	GO:0051179	localization	Biological Process	126
14	GO:0044425	membrane part	Cellular Component	119
15	GO:0050896	response to stimulus	Biological Process	104
16	GO:0044422	organelle part	Cellular Component	82
17	GO:0071840	cellular component organization or biogenesis	Biological Process	74
18	GO:0005215	transporter activity	Molecular Function	53
19	GO:0023052	signaling	Biological Process	51
20	GO:0001071	nucleic acid binding transcription factor activity	Molecular Function	37

The DEGs shared in the 90128- and CN152-infected samples at each time point were also analyzed, and several defense hormone response marker genes were identified. The phenylalanine ammonia-lyase gene (DMG400031457) is homologous to the salicylic acid (SA) marker gene *AtPAL1* in *Arabidopsis thaliana* (Mauch-Mani and Slusarenko 1996), and it was significantly up-regulated at the two time points in response to both 90128 and CN152. Similarly, the jasmonic acid (JA) marker gene jasmonate ZIM-domain protein 1 (DMG400002930) (Wiesel *et al.* 2015), and the abscisic acid (ABA) marker gene homeodomain 20 transcription factor (DMG400003057) (Wiesel *et al.* 2015) were up-regulated in both the 90128- and CN152-infected samples at the two time points. However, the ethylene (ET) marker gene *OSML15* (DMG400003057) (Wiesel *et al.* 2015) and the brassinosteroid (BR) marker gene (DMG400016650) (*AtEXP8* homologs in *Arabidopsis thaliana*) (Malinovsky *et al.* 2014) were both down-regulated in both the 90128- and CN152-infected samples at 24 hpi.

### Analysis of specific DEGs reveals critical genes involved in incompatible or compatible interaction

By comparing DEGs between both time points and treatments, we identified 2,278 and 632 genes at 24 and 72 hpi, respectively, that were specifically up-regulated in the incompatible interaction and 2,504 and 311 genes that were specifically down-regulated. Similarly, in the compatible interaction, 1,594 and 713 genes were specifically up-regulated at 24 and 72 hpi, respectively, and 800 and 597 genes were uniquely down-regulated. All isolate-specific DEGs and their gene expression data are listed in Tables S4 to S11.

We took a conservative approach and identified the top 5% of specific DEGs based on log_2_FC to compare differences in gene expression between susceptible and resistant interactions (Table 5). We found that the expression levels of up-regulated genes in the CN152-infected samples were higher than those in the 90128-infected samples at 24 hpi but lower at 72 hpi. Genes specifically induced in the incompatible interaction include respiratory burst oxidases (DMG400013550, DMG400030390), aminoacyl-tRNA synthetases (DMG402030816, DMG400022055), serine palmitoyl transferases (DMG400009915, DMG400028336, DMG400025070, DMG402008678, DMG400023477), and lignin-forming anionic peroxidases (Novel01106, DMG400027614, DMG400015106). In contrast, *SWEET* genes DMG400011354 (*SWEET9*) and DMG400032771 (*SWEET12-like*), which have previously described as susceptibility-associated genes (Cox *et al.* 2017; Streubel *et al.* 2013), were both significantly specifically up-regulated (64-fold) in the CN152-infected samples at 24 hpi.

**Table 5:** The expression levels of the top 5% of DEGs specifically regulated in response to one isolate

Time points	The *P. infestans* -infected samples	Up- or down-regulated	No. of all specifically regulated DEGs	No. of top 5% of specifically regulated DEGs	Range of Log_2_FC of the top 5% of DEGs	Mean Log_2_FC of the top 5% of DEGs
24 hpi	90128-	up-	2,278	113	4.5181∼9.6887	5.47
	CN152-	up-	1,594	79	5.5692∼12.313	6.95
	90128-	down-	2,504	125	−4.1773∼-11.431	−5.27
	CN152-	down-	800	40	−3.1453∼-6.0518	−4.09
72 hpi	90128-	up-	632	31	5.174∼10.039	6.20
	CN152-	up-	713	35	4.5338∼9.0448	5.91
	90128-	down-	311	15	−3.3937∼-8.7049	−5.27
	CN152-	down-	597	29	−3.2615∼-7.0759	−4.23

### Continuously up-regulated or down-regulated genes contribute more to the defense against P. infestans

Genes continuously up-regulated during the course of *P. infestans* infection were identified based on the following criterion: the FPKM value at each time point was at least twofold higher than the FPKM value at the previous time point. Using this criterion, we identified 73 (Table S12) and 22 (Table S13) genes continuously up-regulated in the 90128-infected and CN152-infected samples, respectively. The continuously up-regulated genes in the 90128-infected samples included those encoding proteins associated with disease resistance: three UDP-glycosyltransferases, two Cytochrome P450s, a basic helix-loop-helix (bHLH) Myc-type transcription factor, an ethylene-responsive transcription factor, an E3 ubiquitin-protein ligase, an LRR receptor-like serine/threonine-protein kinase, and a glucan endo-1,3-beta-glucosidase. The gene DMG400022043 encodes a bHLH Myc-type transcription factor, and its expression was up-regulated about 10-fold at 24 hpi and almost 128-fold at 72 hpi. One of the genes continuously up-regulated in the CN152-infected samples, DMG400019657 encoding UDP-glucosyltransferase, was up-regulated at least 32-fold at 24 hpi and almost 256-fold at 72 hpi. The two *P. infestans*-infected samples shared three continuously up-regulated genes: a subtilisin-like protease gene (DMG400010471), an acetyl-CoA C-acetyltransferase gene (DMG401017380), and a RETICULATA-related protein gene (DMG400032528).

Genes continuously down-regulated during the course of *P. infestans* infection were identified based on the following criterion: the FPKM value at each time point was at least twofold lower than the FPKM value at the previous time point. Using this criterion, we identified 21 (Table S14) and 8 (Table S15) genes continuously down-regulated in the 90128-infected samples and CN152-infected samples, respectively. None of these genes were shared between the two *P. infestans*-infected samples.

### qRT-PCR confirmed the RNAseq results

Ten DEGs were selected for qRT-PCR analysis to validate the results of RNA sequencing. The expression levels of all the selected genes were similar to those from RNA sequencing data, indicating that our transcriptome profiling data were reliable (Figure S1).

## Discussion

### The potato host exhibits distinct expression profiles at the biotrophic and necrotrophic infection stages

The oomycete *P. infestans* has a hemibiotrophic life cycle and exhibits biphasic growth, with an initial biotrophic phase of infection followed by a necrotrophic phase (Ali *et al.* 2014). The time point 72 hpi is considered the start of the necrotrophic phase of the pathogen (Birch *et al.* 2003). In the present study, two time points, 24 and 72 hpi, were chosen as being representative of biotrophy and transition to necrotrophy, respectively. Hierarchical clustering analysis revealed that the changes in gene expression in response to incompatible isolate 90128 and compatible isolate CN152 were different at 24 hpi, but more similar at 72 hpi, suggesting that robust and distinct defense responses are initiated early. Our results contrast the discoveries reported by Tao *et al.* (2003) who found that the degree of the similarity between expression profiles during incompatible and compatible interactions with the bacterial pathogen *Pseudomonas syringae* in *Arabidopsis thaliana* declined at later time points. The fact that we observed the opposite pattern may be attributable to the complex interactions between different host plants and different types of pathogens.

Significant enrichment of KEGG pathways was observed in response to both 90128 and CN152, and these enriched pathways included photosynthesis pathways. This is consistent with the finding of Khorramdelazad *et al.* (2018) who found that a substantial number of genes involved in photosynthesis pathways were differentially expressed during *Ascochyta* infection in lentils. Moreover, we found most of the genes in photosynthesis pathways were down-regulated, irrespective of the outcome of the interaction and this is in agreement with the finding by Attaran *et al.* (2014) and Burra *et al.* (2018) that *P. infestans* infection results in the down-regulation of components of the photosynthetic machinery. *P. infestans* can cause physiological changes in leaves (*e.g.*, lesions and eventual wilting in response to CN152), which affect the photosynthetic capacity of the leaves and therefore would contribute to changes in photosynthesis-related genes.

### 90128 and CN152 activate distinct patterns of defense genes that are characteristic of incompatible and compatible reactions

In our study, we identified more DEGs in the incompatible interaction sample (8,881) than in the compatible interaction sample (7,209), which reflects the different defense responses activated by the two isolates. Given the incompatible and compatible nature of the isolates, we argue that the 8,881 DEGs from the 90128-infected samples represent both basal and *R*-gene mediated responses, while the 7,209 DEGs from the CN152-infected samples are attributed to basal defense responses and the subsequent effects of ETS as well as the pathogen-dependent reprogramming of the plant. Our findings are in agreement with the results of Tao *et al.* (2003) who found that the incompatible interaction between *Arabidopsis thaliana* and *Pseudomonas syringae* led to more changes in mRNA expression than a compatible interaction. We also found that at 24 hpi, a higher number of genes were differentially expressed during the incompatible interaction than during the compatible interaction, while at 72 hpi, substantially more genes were induced during the compatible interaction than during the incompatible interaction. The observations could be explained by unknown avirulence factors in 90128 that induced the ETI reaction, which further activated down-stream signaling pathways involved in defense against the pathogen during the biotrophic phase, leading to increased gene expression.

ETI response in the infected plant is triggered by an oxidative burst and is characterized by an increase in free radicals that leads to PCD (Khorramdelazad *et al.* 2018). In the present study, we found that two respiratory burst oxidase genes (DMG400013550, DMG400030390) were specifically up-regulated in the 90128-infected samples but not in CN152. We also found five serine palmitoyl transferase (SPT) genes that were uniquely up-regulated in the 90128-infected samples. This is consistent with the finding by Avrova *et al.* (1999) that SPTs are involved in cell differentiation and apoptosis. Moreover, two aminoacyl-tRNA synthetase genes (DMG402030816, DMG400022055) were also specifically up-regulated in the 90128-infected samples. Aminoacyl-tRNA synthetase is the receptor of β-Aminobutyric acid, which can induce resistance in many different plant species (Luna *et al.* 2014). Lignin-forming anionic peroxidase genes were also specifically up-regulated in the 90128-infected samples, and these genes may also participate in ETI or the defense response against *P. infestans*. In addition, we found that a bHLH Myc-type transcription factor gene (DMG400022043) was continuously up-regulated after inoculation with 90128, indicating that this gene may play an important role in the incompatible defense response. Consistent with this, a previous study showed that the bHLH transcription factor Myc2 participates in the regulation of plant immunity in tomato (Du *et al.* 2017).

Compared with the molecular mechanisms of incompatible interactions between potato and *P. infestans*, little is known about those underlying compatible interactions. However, some genes associated with susceptibility have been identified. For example, Cox *et al.* (2017) found that *SWEET10* confers susceptibility to bacterial blight in cotton. Streubel *et al.* (2013) found that five rice *SWEET* genes (*OsSWEET11* to *OsSWEET15*) confer susceptibility to *Xanthomonas oryzae*, and they also found that a threshold expression level might be required to support infection. In this study, two *SWEET* genes, DMG400011354 (*SWEET9*) and DMG400032771 (*SWEET12-like*), were specifically highly expressed (both 64-fold) in the CN152-infected samples at 24 hpi, suggesting that these genes may contribute to the susceptibility of 03112-233 to the compatible isolate CN152.

### Complex signal transduction pathways participate in host-pathogen interactions and initiate the defense response

The activation of signaling pathways, most of which are regulated by SA, JA, and ET, induces the expression of defense genes, leading to the production of localized and systemic defenses (Li *et al.* 2016). The SA-mediated signaling pathway is mainly involved in defense against biotrophic pathogens, while JA and ET signaling pathways are, in general, more often activated in response to necrotrophs (van Loon *et al.* 2006). Studies have shown that plant defenses against pathogens are regulated differentially by cross-communicating signal transduction pathways in which SA and JA play key roles. For example, in *Arabidopsis thaliana*, SA-inducible glutaredoxin is involved in suppression of the JA-responsive gene *PDF1.2* (Ndamukong *et al.* 2007), and NPR1 modulates the antagonistic relationship between SA- and JA-dependent defense pathways (Spoel *et al.* 2003).

Yang *et al.* (2018) found that multiple signaling pathways including the SA, JA, and ET pathways were associated with resistance to the super race CN152 in potato genotype SD20. Halim *et al.* (2009) demonstrated that both JA and SA are required for activation of PAMP-induced defense responses in potato. In our study, we found that the SA marker gene DMG400031457 (Mauch-Mani and Slusarenko 1996) and the JA marker gene DMG400002930 (Wiesel *et al.* 2015) were both up-regulated at two time points in response to infection by both 90128 and CN152. However, Smart *et al.* (2003) found that the JA defense response pathway was not induced in a tomato host plant upon infection with *P. infestans*. Restrepo *et al.* (2005) found that genes involved in the JA defense pathway were suppressed in potato during a compatible interaction with *P. infestans*. The conflicting results highlight the complexity of potato-*P. infestans* interactions and more studies are needed to confirm the roles of SA and JA signaling pathways in defense. We also found that the ABA marker gene DMG400000248 (Wiesel *et al.* 2015) was up-regulated in both the 90128- and CN152-infected samples at the two time points, while the ET-induced defense marker gene *OSML15* (DMG400003057) (Wiesel *et al.* 2015) and the BR-induced defense marker gene for expansin (DMG400016650) (Malinovsky *et al.* 2014) were down-regulated in both the 90128- and CN152-infected samples at 24 hpi. Furthermore, the up-regulation of SPTs, which catalyze the first committed step in sphingolipid biosynthesis, indicates that the sphingolipid pathway is also activated during plant defense. We conclude that *P. infestans*-induced defense responses involve the activation of a series of SA, JA, ET, ABA and BR signaling pathways. More detailed studies remain to be conducted further to elucidate the roles of and the cross-talks between components of each of the pathways.

### Molecular mechanisms underlying the incompatible and compatible reactions induced by 90128 and CN152

During the long history of interactions between plants and pathogens, plants have evolved a complete defense system with two components, termed PTI and ETI. This immune system is triggered by recognition of PAMPs or effectors secreted by invading pathogens and the activation of *R* genes. Meanwhile, pathogens can escape recognition by plants by losing or changing PAMPs and by disrupting ETI through the evolution of new effectors (Yang *et al.* 2018). Plants possess basal resistance against pathogens during the early stages of infection, which is overcome, manipulated, or suppressed by these pathogens to allow successful infection and tissue colonization (van Loon *et al.* 2006). In our study, the up-regulation of genes in the *P. infestans*-infected samples at the early stage of infection can be taken as a basic and general defense response to virulent pathogens. The first interaction between the pathogen and the host occurs in the apoplast, where recognition and lysis of the pathogen occur in cases of successful defense (Ali *et al.* 2012). In our study, a large number of glycoside hydrolase genes, which are known to be secreted in the apoplast and to contribute to the primary defense against pathogens (Ali *et al.* 2012), were among the shared DEGs at 24 hpi, suggesting that glycoside hydrolases may play a general role in the primary response to *P. infestans*. Two types of protein kinases, calcium-dependent protein kinases (CDPKs) and leucine-rich repeat receptor-like protein kinases (LRR-RKs), play essential roles in pathogen recognition and early signaling (Khorramdelazad *et al.* 2018), and CDPKs were previously found to be induced by PAMPs in *Psa*-infected kiwifruit (Wang *et al.* 2018). In our study, three genes encoding CDPKs and 17 encoding LRR-RKs were up-regulated in both the 90128- and CN152-infected samples at 24 hpi, suggesting that these *P. infestans* isolates induce common key early signaling genes in potato genotype 03112-233.

The plant *R*-genes can be recognized by specific effectors, resulting in an incompatible interaction (Colton *et al.* 2006). In the present study, according to dRenSeq analysis, no known NLRs (*Rpi_ber*, *Rpi_chc*, *Rpi_R1*, *Rpi_R2*, *Rpi_R2-like*, *Rpi_R3a*, *Rpi_R3b*, *Rpi_R8*, *Rpi_R9a*, *Rpi_tar1*, *Rpi_vnt1.1*, *Rpi_vnt1.3*, *Rpi_Mcq1.1*, *Rpi_Ph-3*, *Rpi_abpt*, *Rpi_amr3*, *Rpi_blb1*, *Rpi_blb2*, *Rpi_blb3*, *Rpi_pta1*, and *Rpi_sto1*) were identified in the potato genotype 03112-233. This suggests that other, so far uncharacterized resistance gene(s), underpin the observed resistance to the isolate 90128. Although the resistance has been overcome by the super race isolate CN152, it may still be of significant practical value to combat against late blight in locations where populations are known to contain no new races that evade the recognition of the resistance gene.
